# Sequence Variation and In Silico Protein Characterization of *γ-TMT* Gene in Mutant Rodent Tuber (*Typhonium flagelliforme* Lodd.)

**DOI:** 10.3390/ijms26157148

**Published:** 2025-07-24

**Authors:** Nesti Fronika Sianipar, Zidni Muflikhati, Reflinur, Muhammad Dylan Lawrie, Dave Mangindaan, Khoirunnisa Assidqi, Chukwunwike Uchenna Enyi, Dwiyantari Widyaningrum

**Affiliations:** 1 Biotechnology Department, Faculty of Engineering, Bina Nusantara University, Jl. K. H. Syahdan No. 9, Jakarta 11480, Indonesia; khoirunnisa.assidqi@binus.edu (K.A.); dwiyantari.widyaningrum@binus.ac.id (D.W.); 2 Food Biotechnology Research Center, Bina Nusantara University, Jl. K. H. Syahdan No. 9, Jakarta 11480, Indonesia; zidnimufli44@gmail.com; 3 Research Center for Applied Botany, Research Organization for Life Sciences and Environment, National Research and Innovation Agency, Bogor 16911, Indonesia; refl001@brin.go.id (R.); muha281@brin.go.id (M.D.L.); 4 Waste-Food-Environmental Nexus Research Interest Group, Bina Nusantara University, Jl. K. H. Syahdan No. 9, Jakarta 11480, Indonesia; 5 Civil Engineering Department, Faculty of Engineering, Bina Nusantara University, Jl. K. H. Syahdan No. 9, Jakarta 11480, Indonesia; 6 Professional Engineering Program Department, Faculty of Engineering, Bina Nusantara University, Jl. K. H. Syahdan No. 9, Jakarta 11480, Indonesia; 7 Department of Food Science and Technology, University of Agriculture and Environmental Science, Umuagwo, Imo, Owerri P.M.B. 1038, Nigeria; nnasedo@gmail.com

**Keywords:** *Typhonium flagelliforme* Lodd., *γ-TMT*, tocopherol, protein prediction, degenerate primer, bioinformatics, computational biology, phylogenetic analysis, polymerase chain reaction

## Abstract

γ-tocopherol is an important antioxidant compound associated with anticancer activity in several plants. This study aimed to analyze the *γ-TMT* (*γ-tocopherol methyltransferase*) gene sequence and predict its protein structure in mutant rodent tuber (*Typhonium flagelliforme* Lodd.) plants. Degenerate primers were designed from homologous sequences in monocot species and used to amplify the *γ-TMT* gene. Amplification of the *γ-TMT* gene was observedin the mutant and the wild-type plants. The amplified region partially covers the *γ-TMT* gene, which has undergone mutations due to a combination of somaclonal variation and gamma irradiation. Sequence analysis revealed notable variations between mutant and wild-type lines, including base substitutions and deletions. Predicted protein structures based on the coding DNA sequence (CDS) revealed notable differences in helix and loop orientation, particularly in the C-terminal domain and central regions of the protein. These structural differences suggest potential links to increased tocopherol biosynthesis or biological activity; however, further experimental validation is required to confirm these functional implications. This study provides foundational insights into the link between the expression of the *γ-TMT* gene and tocopherol biosynthesis and supports the development of specific molecular markers in *T. flagelliforme*.

## 1. Introduction

*Typhonium flagelliforme* Lodd. (family Araceae)—commonly known as rodent tuber—is an indigenous Indonesian plant with significant potential as an anticancer agent, primarily due to bioactive compounds such as fatty acids and sterols. Several studies have reported that these compounds can induce apoptosis and inhibit the proliferation of cancer cells, including breast cancer (MCF-7) cells [1,2,3]. Among these compounds, tocopherol—a member of the vitamin E group—exhibits biological activity contributing to cytotoxic effects against cancer cells [1,2]. Mutation breeding through gamma irradiation has been used to enhance the bioactivity of *T. flagelliforme*. This results in mutant plants that have higher levels of anticancer compounds [4]. Further analysis of these mutants has revealed increased levels of tocopherol and fatty acids, correlating with higher cytotoxic activity [1].

*T. flagelliforme* mutant plants not only contain greater amounts of anticancer compounds than the wild-type, but also demonstrate more effective inhibition of cancer cell growth [4]. The relatively high genetic stability of several mutants has enabled the development of two elite varieties that have been granted Plant Variety Protection (PVP) by the Indonesian Ministry of Agriculture, including the variety ‘Tipobio’ (KB 6-3-3-6 mutant) under registration No. 00489/PPVT/S/2020. Ongoing studies on these mutants have focused on identifying key genes involved in the biosynthetic pathways of anticancer-related metabolites through molecular and biotechnological approaches [1]. Among these pathways, the terpenoid biosynthesis pathway has garnered significant attention due to its role in producing various secondary metabolites with anticancer properties, including tocopherol.

Tocopherol is a bioactive compound within the vitamin E family that plays an essential role in anticancer activity. Its biosynthesis occurs through a sequence of enzymatic reactions in the terpenoid pathway, beginning with geranylgeranyl diphosphate (GGDP), which is converted into homogentisic acid and subsequently into 2,3-dimethyl-5-phytylquinol and γ-tocopherol [5]. The various tocopherol isoforms (α, β, γ, and δ) exhibit distinct biological activities in combating cancer [5]. γ-tocopherol, in particular, has demonstrated synergistic effects with cisplatin in lung cancer cells, enhancing drug cytotoxicity while protecting normal cells from oxidative damage [6]. Moreover, γ-tocopherol is known to inhibit 3-hydroxy-3-methyl-glutaryl-coenzyme A reductase (HMG-CoA reductase), a key enzyme in cholesterol biosynthesis that supports rapid cancer cell proliferation [7]. In vivo studies have further confirmed the efficacy of γ-tocopherol using estrogen-pellet-induced cancer models [8].

Tocopherol biosynthesis genes must be identified using molecular approaches to evaluate *T. flagelliforme* mutants’ anticancer potential. This process involves identifying key genes responsible for tocopherol production and assessing their expression in the mutants. The most significant gene responsible for converting vitamin E into γ-tocopherols is *γ-TMT* (*γ-tocopherol methyltransferase*). This gene plays a crucial role in regulating the metabolism of vitamin E, converting it into its most active form. The activity of *γ-TMT* is essential for maintaining proper cellular function and protecting the body from oxidative stress*. γ-TMT* has been identified in several monocots, including *Oryza sativa* [9]. During this study, *γ-TMT* gene is conserved among monocots. Annotated *γ-TMT* gene sequences from the Araceae family, including *T. flagelliforme*, were not available in public databases. Due to the lack of genomic or transcriptomic data from the Araceae family, degenerate primers were designed based on homologous regions from those well-characterized monocot species. Therefore, degenerate primers were designed based on conserved *γ-TMT* sequences from *O. sativa*, *Z. mays*, and *T. aestivum*. In gene discovery for non-model species, this method is commonly used when close phylogenetic references are not available, allowing for the identification of conserved motifs across distantly related monocots [10]. However, recent research has shown that the gene may play a role in the development of certain traits in other plant families. Further studies are needed to determine whether the *γ-TMT* gene is present in the Araceae family, especially *T. flagelliforme*, and what function it may serve.

Gene sequence analysis plays a central role in uncovering the structure, variation, and functional potential of target genes. Through the analysis of nucleotide sequences, it is possible to detect mutations—such as substitutions, insertions, or deletions—that may influence gene expression or protein activity [10]. Analysis of the *γ-TMT* gene sequence is critical for evaluating the effects of mutagenesis on tocopherol biosynthesis and for assessing genetic homology with other species. The identification and analysis of the *γ-TMT* gene in *T. flagelliforme* is intended to provide baseline data that will be used as a basis for future studies on gene function, the development of molecular markers, and the biosynthesis of bioactive compounds.

In this study, the *γ-TMT* gene was isolated using degenerate primers [11], a strategy that enables the amplification of homologous genes despite sequence variability. Thus, this study aimed to analyze the *γ-TMT* gene sequence related to tocopherol biosynthesis and predict its protein structure in mutant *T. flagelliforme* using a degenerate primer. According to this study, sequence variation and structural differences in encoded proteins were detected and predicted, giving us a preliminary framework for future studies on gene function, tocopherol biosynthesis, and potential bioactive markers. Further empirical validation may contribute to the development of improved germplasm that has enhanced bioactive properties.

## 2. Results

### 2.1. Degenerate Primer Design for the γ-TMT Gene

**Figure 1:**
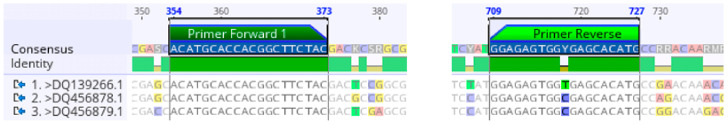
MSA and degenerate primer selection for the *γ-TMT* gene using Geneious v8.1.6 (Biomatters, Ltd., Auckland, New Zealand).

Degenerate primer design for the *γ-TMT* gene was carried out based on three *γ-TMT gene* sequences from monocot plants—*O. sativa* (accession number DQ456878.1), *Zea mays* (DQ456879.1), and *T. aestivum* (DQ139266.1)—retrieved from the GenBank database of the NCBI. During this study, these species were selected because there are no annotated *γ-TMT* gene sequences available from the Araceae family or its closely related families. Furthermore, these monocot species share conserved domains that are suitable for designing degenerate primers. Primers were designed within conserved regions identified through MSA analysis (Figure 1). One forward primer (TMT_F_1) and one reverse primer (TMT_R) were successfully designed (Table 1). The target regions of these primers are part of the CDS, which comprises exonic regions responsible for encoding specific proteins, thus reinforcing the biological relevance of the designed primers [12].

The primers were carefully designed to obtain the key criteria for efficient PCR. The GC content of the primers was over 35%, ensuring strong and stable binding to the target sequence. Additionally, the melting temperature (Tm) difference between the primer pairs was less than 5 °C, supporting optimal annealing efficiency during PCR cycles. The degree of primer degeneracy was maintained below 50 to preserve specificity [13].

BLAST analysis confirmed that the designed primers were specific to monocot species. These primers successfully annealed to the *γ-TMT* gene and related sequences in several monocot species, such as *O. glaberrima*, *Sorghum bicolor*, *Z. mays*, *Phragmites australis*, and *T. aestivum* (Table 2). The primers did not show any matches with dicot species, confirming their specificity to monocots. The length of the PCR products ranged from 309 bp to 3377 bp, reflecting differences in the genomic context and chromosomal locations of the *γ-TMT* gene among the species.

The final result was a pair of degenerate primers that were successfully designed. These primers demonstrated potential for use in *γ-TMT* gene studies across various monocot species, including *T. flagelliforme*. Moreover, these primers can be applied to amplify the *γ-TMT* gene in other monocot species that have not yet been studied. Following their successful design, the primers were utilized for amplification of the *γ-TMT* gene from both mutant and wild-type *T. flagelliforme* DNA samples to evaluate their effectiveness.

### 2.2. γ-TMT Gene Amplification Using Degenerate Primers

**Figure 2:**
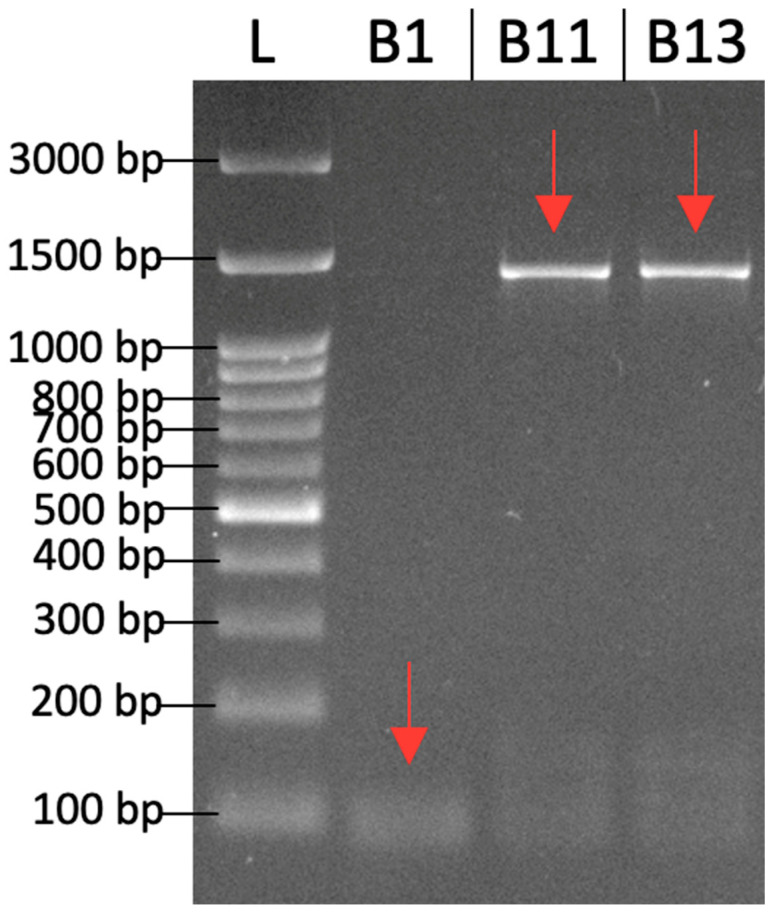
Amplification results using degenerate primers for the *γ-TMT* gene. L: 100 bp DNA ladder (Geneaid Biotech Ltd., New Taipei City, Taiwan). B1 (KB control): wild-type clone. B11 (KB 6-9-3), B13 (KB 6-3-3-6): mutant clones. The red arrows show band clarity.

The amplification results using the designed degenerate primers demonstrated that the primers were capable of producing specific DNA bands from both wild-type and mutant plants (KB 6-9-3 and KB 6-3-3-6), as shown in Figure 2. In the wild-type plant, amplification yielded a DNA band of approximately 100 bp; meanwhile, in the two mutant plants, bands of approximately 1300 bp were observed. The primers designed based on monocot sequences were successfully amplified in all *T. flagelliforme* samples. These findings suggest that the designed degenerate primers are sufficiently sensitive to detect the *γ-TMT* gene despite sequence variations resulting from gamma irradiation.

The successful amplification in both wild-type and mutant samples also reflects the effectiveness of the designed degenerate primers. Sequence analysis of the amplification products was conducted to identify specific nucleotide-level alterations that may be associated with increased tocopherol content—a compound with recognized anticancer properties. Therefore, verification of amplification products through sequencing is an essential step to ensure the identity and integrity of the targeted gene.

### 2.3. Sequencing Verification

The amplification products of the *γ-TMT* gene from both wild-type and mutant *T. flagelliforme* plants were successfully sequenced. A contig was assembled from resulting reads, manually corrected through chromatogram inspection, and trimmed at both ends to eliminate low-quality regions. These verified sequences were subsequently utilized for downstream bioinformatics analyses. BLASTn analysis revealed that the *γ-TMT* gene sequences from the mutant samples exhibited high similarity to homologous genes in various dicotyledonous plant species—including *Carthamus tinctorius*, *C. oxyacanthus*, *Lactuca sativa*, *Helianthus annuus*, *Tabernaemontana elegans*, *G. max*, and *Olea europaea*—as well as in one monocot species (*Elaeis oleifera*), all of which belong to diverse plant families (Table 3). The highest sequence identity was observed with the *γ-TMT* gene of *C. tinctorius* (family: Asteraceae), with identity values reaching 77.90% and highly significant E-values of 8 × 10^−27^ (for sample KB 6-9-3) and 1 × 10^−25^ (for KB 6-3-3-6).

The BLASTn analysis of *γ-TMT* gene sequences from both wild-type and mutant *T. flagelliforme* revealed an average percent identity of approximately 75% when compared to *γ-TMT* gene sequences from various plant species. For the mutant samples, the aligned regions correspond to partial sequences of the *γ-TMT* gene. As a comparison, this gene in *Solanum tuberosum* spans approximately 3967 bp, comprising five introns and six exons, according to data from the NCBI database (Gene ID: 102577815). In contrast, the BLASTn analysis of the wild-type *T. flagelliforme* sequence did not yield any significant similarity to known sequences in the NCBI database. Bioinformatic analyses were conducted to support these findings and provide deeper insight into sequence differences between the mutant and wild-type plants.

### 2.4. Sequence Alignment, Mutation Profiling, and Phylogenetic Analysis of γ-TMT Gene in Mutants

**Figure 3:**
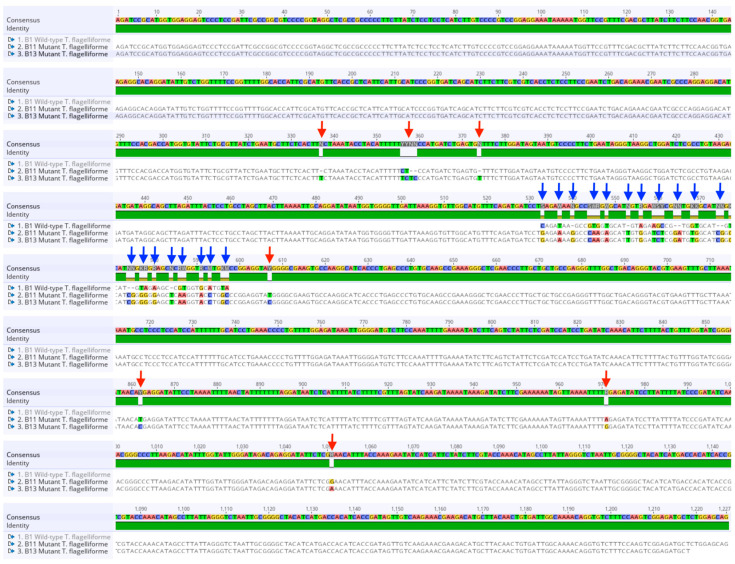
Multiple sequence alignment (MSA) of *γ-TMT* gene sequences in *Typhonium flagelliforme*. Sample B1 represents the wild-type (KB control), while B11 (KB 6-9-3) and B13 (KB 6-3-3-6) are mutant clones. Red arrows indicate point mutations observed between the two mutant sequences. Blue arrows indicate shared mutations between the wild-type and both mutant lines. All mutations are visually annotated and correspond to substitution or deletion events.

Bioinformatics analysis was performed through sequence alignment, aa translation, and phylogenetic tree construction to further investigate the variation of the amplified *γ-TMT* gene between mutant and wild-type *T. flagelliforme*. MSA (multiple sequence alignment) was conducted by comparing the *γ-TMT* sequences of the mutant and wild-type samples. The alignment results revealed that the wild-type sequence aligned with the mutant *T. flagelliforme* sequence, although several nucleotide substitutions and possible insertion-type mutations were observed at specific positions (Figure 3). Specifically, the alignment showed that the wild-type sequence aligned between nucleotide positions 533 bp and 597 bp of the mutant sequence, with a number of mutations detected. These mutations included 13 base substitutions and 4 base deletions (Table 4). In addition, inter-mutant mutations consisting of five nucleotide substitutions and three base deletions were also detected at several positions (Table 5).

A DNA sequence was translated into aa using the *γ-TMT* gene sequence from this study and a reference sequence obtained from a monocot species through BLAST analysis: gamma-tocopherol methyltransferase (g-TMT) mRNA from *E. oleifera* (accession number: EU057617.1) (Figure S1). The translation results showed that the *T. flagelliforme* sequence aligned starting from base position 158 bp of the reference gene, indicating that the *γ-TMT* mutant sequence obtained corresponds to the exon region beginning at that position. Furthermore, the aa translation analysis revealed a difference in the number of amino acids between the two mutants: the KB 6-3-3-6 mutant produced 406 aa, while the KB 6-9-3 mutant produced 407 aa. Meanwhile, the wild-type only translated into 19 aa. The identified exon region consisted of 354 aa in KB 6-3-3-6 and 355 aa in KB 6-9-3, while the amplified region in the wild-type was entirely composed of exon sequences. The observed differences in amino acid number and alignment patterns indicate variation in the translated regions among wild-type and mutant plants.

**Figure 4:**
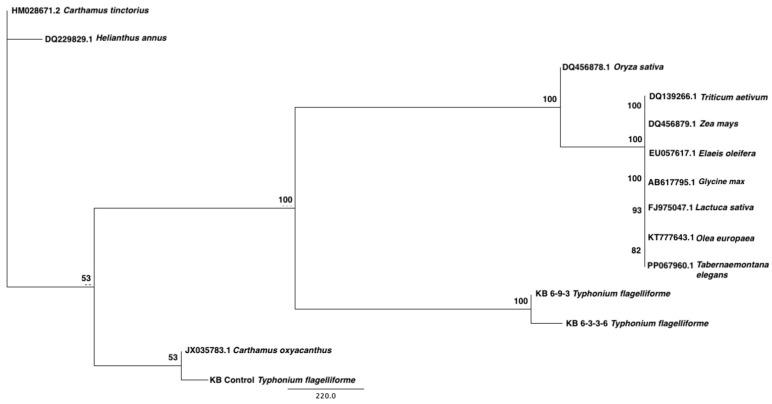
Phylogenetic tree construction of *γ-TMT* gene sequences from wild-type and mutant *T. flagelliforme*, along with several other species. Bootstrap values (e.g., 100, 93, 82, 53) are shown at branch nodes and indicate the percentage of times a given branch was supported in 1000 bootstrap replicates with 100 indicating maximum support and values below 70 generally considered low. The scale bar represents the number of nucleotide substitutions per site.

The phylogenetic analysis aimed to assess the evolutionary relationships between *γ-TMT* sequences in *T. flagelliforme* and those in other plant species, providing a comparative framework. These results can be strengthened by conducting phylogenetic analysis to determine the relationship between the identified sequences and *γ-TMT* gene sequences from other plant species. Thus, a phylogenetic tree of the *γ-TMT* gene from *T. flagelliforme* and various plant species was constructed (Figure 4). The results show that the *γ-TMT* sequence from wild-type *T. flagelliforme* (family: Araceae) is closely related to that of *C. oxyacanthus* (family: Asteraceae; accession JX035783.1), while the mutant sequences grouped separately. These patterns suggest localized sequence variation in the *γ-TMT* gene due to induced mutagenesis [4]. The amplified sequence obtained using degenerate primers was successfully identified as a CDS at the 158th amino acid position.

### 2.5. γ-TMT Protein Structure Prediction in Mutants

Our in silico protein structure predictions were based on coding sequences from two mutant lines of *T. flagelliforme*, which produced approximately 1300 bp amplicons. A homolog search for the tocopherol methyltransferase gene through the NCBI database revealed that the γ-TMT protein sequence from mutant KB 6-9-3 shares similarities with several proteins from various plant species (Table 6). The highest sequence identity was 73.61%, corresponding to two proteins: a hypothetical protein from *Drosera rotundifolia* and gamma-tocopherol methyltransferase from *Theobroma cacao*. A hypothetical protein is a protein that is predicted based on genomic data, but which has not yet been functionally characterized [14].

Although the sequence identity is relatively high, the query coverage is only 21%. Thus, only a small part of the entire protein structure length has similarity. It can be attributed to the significant taxonomic differences between *T. flagelliforme* (family: Araceae) and *T. cacao* (family: Malvaceae). Meanwhile, the BLASTp search of the *γ-TMT* sequence from the KB 6-3-3-6 mutant did not show significant similarity with any protein sequences in the NCBI database.

**Figure 5:**
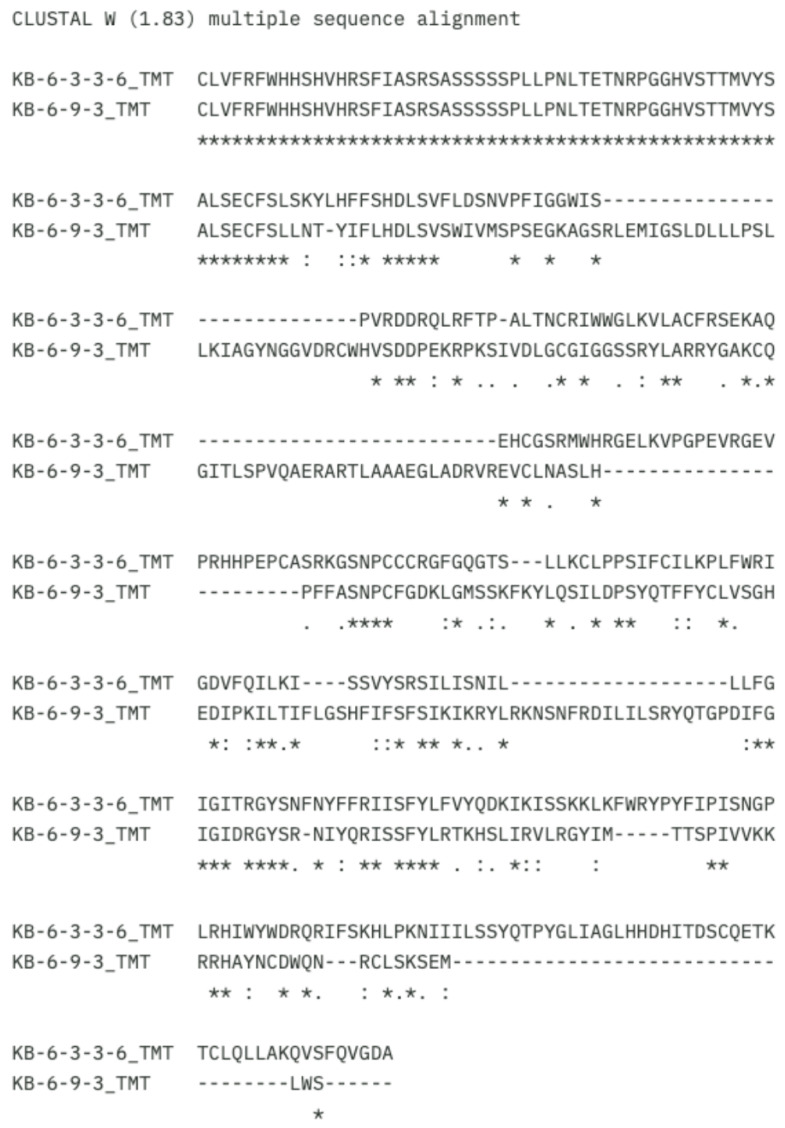
MSA results for CDS aa protein sequences. KB-6-3-3-6 and KB-6-9-3: mutant clones. The asterisk (*) symbol indicates identical sequence similarity. The colon (:) symbol represents strong (conservative) similarity. The period (.) symbol indicates weak similarity. The dash (-) symbol denotes a gap. Blank spaces indicate no similarity.

The MSA results for the CDS aa protein sequences show significant variation between mutants KB 6-3-3-6 and KB 6-9-3 (Figure 5). Identical similarities were found only at a few limited positions, marked with an asterisk (*). These positions represent residues that are identical in both mutants, which are generally located in regions that may be conserved or structurally important. Meanwhile, most regions exhibit differences, indicated by colons (:) or periods (.), which represent conservative and weak substitutions, respectively. Gaps (-) can also be observed in the KB 6-9-3 mutant, particularly from the middle to the end of the sequence (Figure 5). These gaps indicate deletions in this mutant, which may influence the overall structure and function of the protein. In the context of protein structure, aa deletions or insertions can lead to changes in important binding domains or affect proper protein folding [15].

**Figure 6:**
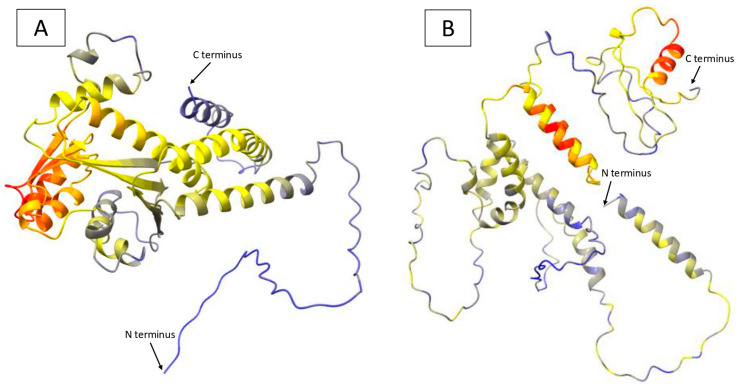
Predicted protein structure of γ-TMT in *T. flagelliforme* mutants. (**A**) γ-TMT protein structure in the KB 6-9-3 mutant. (**B**) γ-TMT protein structure in the KB 6-3-3-6 mutant. Blue (pLDDT > 90): high prediction confidence. Yellow (pLDDT 50–70): medium prediction confidence. Orange–red (pLDDT < 50): low prediction confidence.

The three-dimensional structure prediction for *γ-TMT* proteins from two *T. flagelliforme* mutants revealed overall differences in protein folding configurations (Figure 6). The color in each structural model represents the pLDDT (predicted Local Distance Difference Test) score, which indicates the confidence level of the structure predicted using AlphaFold/ColabFold. The *γ-TMT* structure in the KB 6-9-3 mutant displays a high pLDDT score (blue), suggesting a reliable prediction with potential structural stability (Figure 6A). On the other hand, the *γ-TMT* structure in the KB 6-3-3-6 mutant shows a medium pLDDT score (yellow), indicating moderate prediction confidence and possible structural variability (Figure 6B).

**Figure 7:**
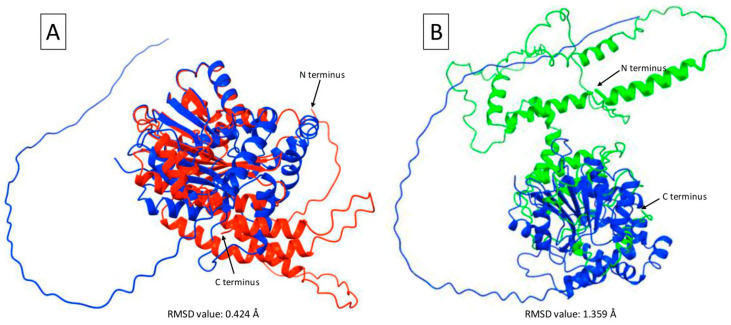
Superimposition of protein structures predicted using AlphaFold2. (**A**) Mutant KB 6-9-3 (red) compared with the γ-TMT protein model (EOY10208.1; blue); RMSD = 0.424 Å. (**B**) Mutant KB 6-3-3-6 (green) compared with the γ-TMT protein model (EOY10208.1; blue); RMSD = 1.359 Å. Structural differences can be observed in the KB 6-3-3-6 mutant (green), particularly in the C-terminal domain.

The predicted γ-TMT protein structure was superimposed using the aa sequences from the CDS of the γ-TMT protein in *T. flagelliforme* mutants to observe structural changes resulting from substitutions and base deletions, particularly in the tertiary structure of the mature protein. The modeling results reveal significant changes in the tertiary structure of γ-TMT between mutants KB 6-9-3 and KB 6-3-3-6 compared with the reference protein model (Figure 7). The KB 6-9-3 mutant shows excellent superimposition with the protein model (Figure 7A), indicating high structural similarity. In contrast, the KB 6-3-3-6 mutant exhibits suboptimal superimposition (Figure 7B), reflecting considerable structural differences. The γ-TMT protein structure model used is from *T. cacao* (accession number: EOY10208.1), based on BLASTp alignment, and showed that the *γ-TMT* sequence from *T. cacao* has the highest similarity to the *γ-TMT* sequence from the *T. flagelliforme* mutants.

Further structural analysis has identified notable differences in the C-terminal domain of the KB 6-3-3-6 mutant compared with the γ-TMT protein model. These differences were quantified using Root Mean Square Deviation (RMSD) analysis. The KB 6-3-3-6 mutant exhibited a higher RMSD value of 1.359 Å, whereas the KB 6-9-3 mutant showed a lower RMSD value of 0.424 Å.

On the other hand, the theoretical average molecular weight of the *γ-TMT* protein from mutants KB 6-9-3 and KB 6-3-3-6 did not show significant differences, at 38.53 kDa and 38.56 kDa, respectively (Figure S2). Similarly, the theoretical pI values for both mutants were also similar, at 9.66 and 9.67, respectively (Figure S2). The results of this study indicate that significant structural differences in the protein do not necessarily affect the pI and molecular weight values substantially.

## 3. Discussion

### 3.1. Primer Efficiency and γ-TMT Gene Amplification

The successful amplification of the *γ-TMT* gene in both wild-type and mutant *T. flagelliforme* plants demonstrates the effectiveness of the degenerate primers designed from conserved monocot sequences. Although the amplicon size differed—approximately 100 bp in the wild-type and 1300 bp in the mutants—the primers successfully amplified the *γ-TMT* gene in all genotypes. The differences in amplicon size between the wild-type and mutants are likely due to genetic alterations induced by gamma irradiation, which can cause insertions, deletions, or mutations in the primer binding sites or within the amplified region [4].

The *γ-TMT* gene is one of the key genes involved in the tocopherol biosynthesis pathway, a compound with high antioxidant activity and a crucial biological role in protecting cells against oxidative stress [5,9]. Tocopherol also has biological activity beyond antioxidation, including potential anticancer effects [16,17]. In mutant *T. flagelliforme* plants, variations in tocopherol content are suspected to be related to gamma irradiation treatment [4]. Gamma irradiation can induce genetic variations that may affect the structure and position of genes, including the *γ-TMT* gene, without necessarily abolishing their function [18]. Therefore, the successful detection of the *γ-TMT* gene in both wild-type and mutant plants suggests that this gene is stable and essential, even after mutagenesis. The primers designed in this study showed high specificity for the target sequence. This highlights the potential utility of these primers for future applications in other Araceae species, despite the lack of close phylogenetic reference data.

Furthermore, this study provides new insights into the impact of gamma irradiation on plant genome structures. Although gamma irradiation can generate genetic variations [4,19], essential genes such as *γ-TMT* can still be isolated from mutant plants. This research not only confirms the presence of the *γ-TMT* gene in mutants but also provides initial evidence supporting a possible link between *γ-TMT* gene variation and enhanced tocopherol content in mutant *T. flagelliforme* plants. The KB 6-3-3-6 mutant, for instance, has been found to contain a higher level of tocopherol than the wild-type plant [4]; however, the direct functional relationship between *γ-TMT* gene variation and tocopherol content requires further investigation. The presence of a 1300 bp amplification band in the mutant may reflect underlying genomic alterations associated with changes in tocopherol biosynthesis.

Previous studies have shown increased tocopherol and fatty acid content in gamma-irradiated *T. flagelliforme* mutants, which may contribute to their enhanced cytotoxic properties [1,4]. However, the specific contribution of *γ-TMT* variation to these effects requires further investigation. Other gene identification studies using similar strategies have been reported in non-model plants under mutagenesis, such as in tomato for the phytoene synthase gene [20], and in maize for the *γ-TMT* gene [21].

Only one individual per genotype was sequenced in this study, which may not fully capture variation within genotypes. However, the PCR amplification was repeated to ensure band consistency. The two mutant lines analyzed were independently derived and exhibited stable phenotypic traits. The presence of *γ-TMT* in both wild-type and mutant samples confirms its stability post-irradiation. Further studies with biological replicates and functional assays are needed to fully elucidate its role in tocopherol biosynthesis.

### 3.2. Sequence Variation and Bioinformatics Analysis

The BLASTn results revealed significant sequence similarity between the mutant *γ-TMT* gene fragments and those of several dicotyledonous species. High identity values and extremely low E-values—for example, 8 × 10^−27^—indicates high statistical significance, suggesting that the similarity is not due to random chance but, rather, reflects a true evolutionary or functional relationship. In bioinformatics, a percentage identity greater than 70% is generally considered sufficient to indicate genetic conservation and homology, suggesting that the genes in question likely share a similar function or originate from a common evolutionary ancestor [22]. In contrast, no significant similarity was found for the wild-type sequence. This discrepancy may be due to the limited genomic representation of *T. flagelliforme* and the Araceae family in general within public sequence repositories. Alternatively, it is possible that the amplified wild-type fragments represent non-conserved or incomplete regions of the *γ-TMT* gene. The identification of a *γ-TMT* homolog in the mutant supports its assignment as a *γ-TMT* gene fragment from *T. flagelliforme*. The partial sequence may contain unique features compared to known *γ-TM*T genes, highlighting its potential relevance for future studies on tocopherol biosynthesis in medicinal plants.

The multiple sequence alignment of aa sequences revealed significant differences between the wild-type and mutant samples, indicating that rearrangements had occurred in the *γ-TMT* gene sequence of the mutants. These changes affected the aa translation results, likely due to a combination of somaclonal variation and gamma-ray irradiation treatment [4]. Although band sizes were similar, sequence-level differences were detected among the mutants.

In this study, the most frequently observed mutations in the mutant *γ-TMT* gene were deletions and substitutions, which altered amino acid reading frames and may impact protein structure and function [10,23]. Similar mutations have been shown in other plant genes, such as *PAL* (*Phenylalanine Ammonia-Lyase*), to affect enzyme function and metabolite biosynthesis [24]. It is important to note that this study focused on sequence-level variations and did not investigate larger chromosomal rearrangements such as gene translocation or inversion. However, the possible role of larger genomic rearrangements cannot be ruled out and warrants future investigation. Mutations may influence the biosynthesis of tocopherol and other related compounds through gene expression changes. Therefore, this study provides a critical foundation for further gene expression analysis related to the molecular mechanisms underlying the enhanced production of bioactive compounds and increased cytotoxic activity in mutant plants.

Previous research has successfully identified partial sequences of the *lectin* gene—one of the genes associated with anticancer activity—in both wild-type and mutant *T. flagelliforme* plants [25]. These findings were further developed into two gene-specific molecular markers—namely, Single-Nucleotide Amplified Polymorphism (SNAP) and the Amplification-Refractory Mutation System (ARMS)—which can differentiate between wild-type and mutant *T. flagelliforme* plants [25]. This study revealed a segment of another gene associated with anticancer activity—the *γ-TMT* gene—in both mutant and wild-type plants. Our findings provide a foundation for exploring genetic diversity based on genes involved in anticancer compound biosynthesis.

### 3.3. Protein Modeling and Structural Changes

To characterize the *γ-TMT* gene sequence obtained in this study, a reference gene was required for translation and multiple sequence alignment (MSA). The gamma-tocopherol methyltransferase (g-TMT) mRNA from *E. oleifera* (accession number: EU057617.1) was selected as a reference for translation and MSA due to its clear start codon. Although it is a partial CDS, this gene includes a portion of the coding region that encodes the γ-TMT protein.

Significant differences in amino acid composition were observed between the wild-type and mutant sequences, primarily due to deletions in both types. These changes may alter the protein’s functional domains and potentially affect its enzymatic activity [10]. *γ-TMT* is involved in the tocopherol biosynthesis pathway, essential for vitamin E production [9]. Therefore, structural changes in the *γ-TMT* gene and protein caused by mutations may directly affect the accumulation of tocopherol-related compounds. A full-length sequence obtained from both genotypes is required to fully analyze the structural and functional differences between the two genotypes. Furthermore, these data can also serve as a foundation to develop specific molecular markers for the *γ-TMT* gene associated with anticancer compounds.

The phylogenetic divergence between the *γ-TMT* sequences from mutant and wild-type *T. flagelliforme* may be attributed to gamma irradiation, which can introduce random mutations affecting both coding and regulatory regions. These mutations could have caused enough sequence divergence to alter the clustering pattern in the phylogenetic tree. Additionally, the observed close relationship between the *γ-TMT* gene of *T. flagelliforme* and those from distantly related species (e.g., *E. oleifera* and *G. max*) may be due to conserved functional domains in the methyltransferase gene family, which are maintained across evolution despite taxonomic differences. Such conservation is common in enzymatic proteins involved in essential metabolic pathways like tocopherol biosynthesis [26]. This observation highlights the complexity of using phylogenetic reconstruction in non-model species with partial gene sequences and underscores the need for full-length sequence analysis to better resolve evolutionary relationships.

In addition to producing different amplicon sizes—approximately 100 bp in the wild-type and 1300 bp in the mutant—the amplification results suggest that mutagenesis induced sequence variations affecting *γ-TMT* structure. These variations may influence the biosynthesis of tocopherol-related anticancer compounds. Overall, the findings highlight how induced mutations can reshape gene structure and potentially alter biological function, thereby providing valuable insights for functional genomics and molecular breeding in medicinal plants. CDS-derived sequences enabled structure prediction, although domain-level functional conservation was not specifically assessed in this study [27,28]. Therefore, further investigation is needed to determine whether key catalytic or substrate-binding domains are preserved across species.

These functional insights must also be considered in the context of available genomic data; this is notable due the limited data on gamma-tocopherol methyltransferase protein sequences in the Araceae family, particularly in the *Typhonium* genus. Most of the homologous proteins identified in this study were recognized as γ-tocopherol methyltransferase, an enzyme involved in tocopherol (vitamin E) biosynthesis. These results suggest that the *γ-TMT* sequence from the *T. flagelliforme* mutant is involved in the tocopherol biosynthetic pathway. Furthermore, the presence of a hypothetical protein with a similar sequence identity indicates that there are still many proteins from non-model species that have not been functionally annotated [29]. The very small E-value (≤6 × 10^−23^) strengthens the hypothesis that the sequence similarity is not random but, rather, biologically significant. Further validation of the function and specificity of this gene can be performed through gene expression analysis or enzyme activity assays.

Protein modeling showed topological differences between the two mutants, particularly in the helices and loops near the C-terminal and central domains. These variations likely result from mutations that alter the spatial conformation of γ-TMT, potentially affecting enzymatic function. Changes near conserved or active sites may disrupt the biosynthesis efficiency of bioactive compounds such as tocopherol [30].

Structural changes due to mutations can affect protein functions, particularly if they occur around the active domain or substrate-binding sites. In many transferase enzymes, including tocopherol methyltransferase, the stability of helices and loops is crucial to ensuring optimal binding with cofactors, ligands such as S-adenosylmethionine (SAM), and vitamin E intermediates [26]. Therefore, significant structural shifts in mutant KB 6-3-3-6 may alter the enzyme’s efficiency or disrupt its enzymatic function. The structure prediction was generated using AlphaFold2, a deep learning-based system that utilizes transformer architecture and the PDB structure database to generate high-quality protein models [27,28]. Treating the pLDDT values as local confidence indicators, these results suggest that the *γ-TMT* structures from mutants KB 6-9-3 and KB 6-3-3-6 are suitable for downstream analyses, including the generation of functional hypotheses and molecular ligand design.

The KB 6-3-3-6 mutant shows more significant tertiary structure differences than the KB 6-9-3 mutant, likely due to substitutions and deletions in the CDS of the *γ-TMT* aa sequence. Substitutions and deletions affected helices and loop regions, which are essential for protein stability and molecular interactions [29,31]. These structural alterations may underlie the differences in γ-TMT activity between the mutant lines. Similar structural changes in other plant-derived proteins have been linked to altered anticancer activity, including effects on mitotic arrest and apoptosis in cancer cell lines [32,33,34,35].

Analysis of the RMSD data was conducted to assess the structural divergence between mutants. RMSD quantifies the average conformational deviation between two protein structures; however, it should be interpreted cautiously, as it does not directly indicate protein stability without further energetic or molecular dynamics analyses [36,37]. Given these results, the KB 6-9-3 mutant’s protein structure appears to be more similar to the reference model than that of KB 6-3-3-6. Nevertheless, further experimental validation is needed to confirm any functional or stability implications. Structural changes occurring in the C-terminal domain are known to play a crucial role in enzyme stability and activity, potentially affecting the efficiency of γ-tocopherol conversion to α-tocopherol. This finding is consistent with the study by [38], who emphasized that the structural integrity of the *γ-TMT* enzyme is vital for the vitamin E biosynthesis pathway in plants.

The less optimal superimposition and apparent lower structural similarity observed in the KB 6-3-3-6 mutant may be attributed to the phylogenetic differences between *T. flagelliforme* (Araceae family) and *T. cacao* (Malvaceae family), which served as the template for the *γ-TMT* protein model. This suggests that the structure of *γ-TMT* from *T. flagelliforme* may possess unique characteristics distinguishing it from other species. These characteristics may affect its specific functions, including its role in the tocopherol metabolic pathway and its potential biological activities, such as anticancer effects [33,38].

In addition to tertiary structural differences, physicochemical properties, such as isoelectric point (pI) and molecular weight (Mw), were also analyzed (Figure S2). Each aa has a different pI, which occurs when the pH of an aa reaches a neutral condition (zwitterion) due to the addition or removal of protons in an acid–base reaction. At this point, protein solubility can decrease, leading to precipitation and aggregation. The results of this study indicate that significant structural differences in the protein do not necessarily affect the pI and molecular weight values substantially.

These observations are consistent with previous reports by Savino et al. [10], who found that mutations (such as substitutions and deletions) can lead to changes in amino acid translation, subsequently altering the protein structure. Mutations in the sequence of KB 6-3-3-6 mutant may alter the protein structure [39]. Several studies have reported that mutation locations do not always directly impact functional domains, but others have shown a loss or alteration of function at the functional level [39]. The results of this study suggest that a combination of somaclonal variation and gamma-ray irradiation treatment may induce significant changes in the mutant protein topology [4,19]. These structural changes may affect gene expression and biological activity in vitro against various types of cancer cells [40].

Although previous studies have reported on the general function of *γ-TMT* in tocopherol biosynthesis, we provide the structural variations of *γ-TMT* protein, specifically, in *T. flagelliforme* mutants induced by gamma irradiation and somaclonal variation. Our structural insights enhance our understanding of gene variations within the Araceae family. They also lay the groundwork for improved bioactive compound production through molecular breeding and biotechnological advancements. Thus, this study contributes valuable knowledge that could aid the development of functional foods and phytopharmaceuticals benefiting society by supporting cancer prevention and therapy.

Overall, the findings of this study strengthen the hypothesis that the combination treatment of somaclonal variation and gamma-ray irradiation alters the structure of the *γ-TMT* gene at the nucleotide, aa, and protein levels without completely abolishing its function [41]. According to previous studies, the KB 6-3-3-6 mutant of *T. flagelliforme* has higher tocopherol content compared to the wild-type and other mutants [4]. These findings provide a hypothesis that *γ-TMT* gene structural changes may affect tocopherol biosynthesis; however, direct functional validation is required to confirm this relationship. While the KB 6-3-3-6 mutant has previously been shown to contain higher tocopherol content, this study does not directly link *γ-TMT* mutations with anticancer compound biosynthesis, and the proposed relationship remains hypothetical. This analysis provides an important foundation for further investigation of the relationship between mutations and the increased tocopherol content in *T. flagelliforme*, as well as the production of bioactive compounds with anticancer potential. The *γ-TMT* gene may serve as a candidate target for future molecular marker development and functional validation, particularly in breeding or genetic engineering programs aimed at enhancing the medicinal value of this species.

## 4. Materials and Methods

### 4.1. Plant Genetic Materials

**Figure 8:**
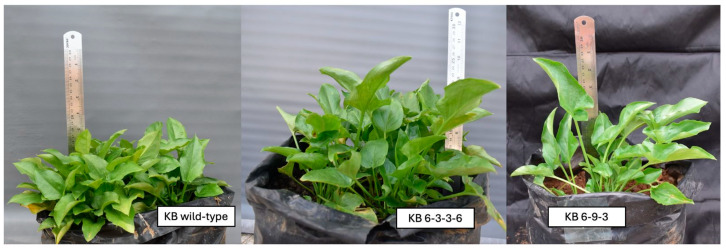
The morphological characteristics of the wild-type and mutant *T. flagelliforme* plants (KB 6-3-3-6 and KB 6-9-3).

Three genotypes of *T. flagelliforme* were used in this study, including a wild-type plant as a reference and two mutant lines, KB 6-3-3-6 and KB 6-9-3. The two mutant plants were selected based on their stable phenotypic characteristics and distinct mutational backgrounds (high tocopherol and fatty acid contents as well as different leaf and stem morphology). The morphological characteristics of the wild-type and mutant plants (KB 6-3-3-6, KB 6-9-3,) are shown in Figure 8. All plant materials were obtained from the collection maintained by Bina Nusantara University (Jakarta, Indonesia) in 2021. *T. flagelliforme* accessions used originated from Bogor, Indonesia, while the mutant lines were developed through gamma irradiation in previous studies [4].

### 4.2. Genomic DNA Isolation and Quality Test

Fresh leaf tissues were collected from one wild-type and two mutant individuals of *T. flagelliforme* (KB 6-3-3-6 and KB 6-9-3), maintained at the Bina Nusantara University collection (2021, Jakarta, Indonesia). The plants originated from the Bogor accession. Genomic DNA was isolated using a modified CTAB (cetyltrimethylammonium bromide) method. Leaf samples were frozen in liquid nitrogen and ground into a fine powder, followed by the addition of 1000 µL of extraction buffer. The mixture was transferred into a 2 mL microcentrifuge tube and incubated in a water bath at 65 °C for 45 min, with homogenization every 15 min.

After incubation, 800 µL of chloroform–isoamyl alcohol solution (24:1, *v*/*v*) was added to the tube and mixed thoroughly. The mixture was then centrifuged at 13,500 rpm for 10 min. Subsequently, 400 µL of the supernatant was transferred into a new 1.5 mL microcentrifuge tube, and 500 µL of cold isopropanol and a 0.1× volume of sodium acetate was added. The tube was tightly closed and gently mixed to enable DNA precipitation at room temperature for 10 min, followed by centrifugation at 13,500 rpm for another 10 min.

The resulting DNA pellet was dissolved in TE buffer (Tris-EDTA) and treated with RNase (10 mg/mL) to remove RNA contamination. The mixture was gently mixed and incubated at 37 °C for approximately 30 min, then centrifuged again at 13,000 rpm for 10 min. The supernatant was discarded, and the pellet was washed with 600 µL of 95% ethanol. After incubation at −20 °C for 45 min, the sample was centrifuged at 13,500 rpm for 10 min to remove the ethanol. The dried DNA pellet was resuspended in 200 µL of TE buffer. In this study, 1.4% agarose gel electrophoresis was used to verify genomic DNA. It was stored under optimal conditions as described by Sianipar et al. [12]. A spectrophotometer was used to reassess DNA concentration before amplification.

### 4.3. Primer Design and Amplification of γ-TMT

Degenerate primer design was performed based on *γ-TMT* gene sequences from several monocot plants retrieved from the GenBank database of the National Center for Biotechnology Information (NCBI) (https://www.ncbi.nlm.nih.gov/ accessed on 3 May 2025). All sequences were downloaded in FASTA format and aligned using two multiple sequence alignment (MSA) methods: ClustalW implemented in Geneious v8.1.6 (Biomatters, Ltd., Auckland, New Zealand) and MUSCLE in MEGA v11 (Pennsylvania State University, State College, PA, USA). Primers were designed based on conserved regions identified within the coding DNA sequences (CDSs). One primer pair was selected with an emphasis on its efficiency and specificity to the target gene. Only one pair of degenerate primers was used to amplify the *γ-TMT* gene. Primer binding sites were identified and visualized on the target sequence. The designed primers were tested for specificity using the Primer-BLAST (version 4.1.0) tool on the NCBI website (https://www.ncbi.nlm.nih.gov/tools/primer-blast/ accessed on 3 May 2025) to ensure alignment with the *γ-TMT* gene and to minimize the possibility of off-target binding.

PCR amplification was performed using the designed degenerate primers and genomic DNA from wild-type and mutant *T. flagelliforme*. Amplification was performed using MyTaq™ HS Red Mix (Bioline Ltd., London, UK) with 20 ng of template DNA, according to the manufacturer’s instructions. The PCR conditions were as follows: initial denaturation at 94 °C for 4 min, followed by 40 cycles of denaturation at 94 °C for 30 s; annealing at 66.1 °C for the wild-type sample and 63.8 °C for both mutant samples for 20 sec; extension at 72 °C for 50 s; and a final extension at 72 °C for 5 min. Various annealing temperatures were used to optimize band clarity. Both genotypes produced bands of expected size when amplification was performed at 66.1 °C. Mutant bands were less apparent. By reducing the annealing temperature for mutant samples to 63.8 °C, we achieved better sequencing quality. The resulting amplicons were visualized on a 2% agarose gel. DNA bands were then excised and sent to 1st BASE (Malaysia) for sequencing. PCR products from wild-type (KB wild-type), KB 6-3-3-6 (code B13), and KB 6-9-3 (code B11) were sequenced and labeled. PCR products from the wild-type and mutant samples were sequenced in both forward and reverse directions. Sequence reads were assembled into contigs and manually verified through chromatogram inspection to ensure data accuracy and reliability. The obtained sequences were subsequently verified using BLAST analysis. The amplified *γ-TMT* gene sequences from the two mutant lines, KB 6-9-3 and KB 6-3-3-6, have been submitted to the GenBank database under accession numbers PV791201 and PV791202, respectively.

### 4.4. Bioinformatics Analysis

The bioinformatics analysis in this study included the characterization of *γ-TMT* gene sequences and translation amino acids (aa) to compare the *γ-TMT* gene sequence of wild-type *T. flagelliforme* with its mutant variants. Sequencing reads were assembled into contigs and manually curated by inspecting chromatograms. Trimming was performed at both ends (5′ and 3′) to remove low-quality base calls. These curated sequences were then used for BLAST and multiple sequence alignment (MSA) analyses. MSA was conducted using the ClustalW method within Geneious v8.1.6 software (Biomatters, Ltd., Auckland, New Zealand). The aligned sequences were then used to construct a phylogenetic tree using the Maximum Likelihood (ML) method with 1000 bootstrap replicates in MEGA v11 (Pennsylvania State University, State College, PA, USA). The optimal nucleotide substitution model was selected using the Akaike Information Criterion (AIC) prior to tree construction. Sequence differences were recorded and categorized as substitution or deletion mutations.

### 4.5. γ-TMT Protein Structure Prediction of Mutant Rodent Tuber

γ-TMT protein analysis was based on the coding DNA sequence (CDS) of the *γ-TMT* gene from mutant *T. flagelliforme*, with an approximate length of 1064 base pairs (bp) for KB 6-9-3 and 1059 bp for KB 6-3-3-6. Gene sequences were translated into proteins using the Expasy Translate tool (https://web.expasy.org/translate/ accessed on 05 March 2025). Amino acid sequence alignment was performed using the Clustal Omega (version 1.2.4) tool provided by EMBL-EBI (https://www.ebi.ac.uk/jdispatcher/msa/clustalo accessed on 05 March 2025). Protein BLAST analyses were then conducted using the UniProt (https://www.uniprot.org/blast accessed on 05 March 2025) and NCBI platforms to identify potential changes in protein types resulting from mutations. Aa sequences were used to predict protein structures using AlphaFold2/ColabFold v1.5.5. Protein structure files (PDB format) were obtained using γ-TMT as the model protein and visualized using UCSF ChimeraX. The molecular weight and isoelectric point (pI) of the tertiary protein structures were estimated using the Expasy Compute pI/Mw tool (https://web.expasy.org/compute_pi/ accessed on 05 March 2025).

## 5. Conclusions

This study successfully analyzed the *γ-TMT* gene sequences in both mutant and wild-type *T. flagelliforme* plants and predicted structural differences in the encoded proteins through the design of a pair of degenerate primers. A PCR amplification produced a DNA band of approximately 1300 bp in mutant plants and around 100 bp in wild-type plants. Mutations and deletions were detected between wild-type and mutant lines, including thirteen base substitutions and four deletions, and five substitutions and three deletions between mutant lines. A translation of amino acids showed that mutant proteins encoded (406–407 aa) as opposed to wild-type proteins (19 aa). AlphaFold2 structural modeling revealed significant changes in conformation, particularly in the C-terminal loops and helices. A direct link to the biosynthesis of anticancer compounds remains hypothetical, although there may be a connection with increased tocopherol content observed previously in mutants. Thus, further experimental validation is required. Nonetheless, these results support the development of molecular markers to identify *T. flagelliforme* metabolites that produce bioactive compounds. This research provides a valuable basis for future research. These markers could help identify specific compounds with therapeutic potential, facilitating targeted studies on their pharmacological effects.

## Figures and Tables

**Table 1:** Designed degenerate primers for the *γ-TMT* gene.

Gene	Primer Id	Sequences Primer (5′→3′)	Tm (°C)
*γ-TMT*	TMT_F_1	ACATGCACCACGGCTTCTAC	64.6
	TMT_R	CATGTGCTCRCCACTCTCC	65.2–67.6

Note: The red color indicates degenerate bases. Degenerate base code R = G or A. F: forward; R: reverse.

**Table 2:** BLAST results for the designed *γ-TMT* gene primers.

No	Accessions	Monocots Species	Descriptions	Product Size
1.	CP137583.1	*Eragrostis tef* cultivar Dabbi	Chromosome 1B	1273 bp
2.	CP137582.1	*Eragrostis tef* cultivar Dabbi	Chromosome 1A	3377 bp
3.	XM_052289477.1	*Oryza glaberrima*	Probable tocopherol O-methyltransferase, chloroplastic (LOC127764584), mRNA	368 bp
4.	OK032047.1	*Oryza punctata*	Isolate RWG-480 *gamma-tocopherol methyl transferase* (*gammaTMT*) gene, complete cds	1688 bp
5.	OK032037.1	*Oryza longistaminata*	Isolate RWG-470 *gamma-tocopherol methyl transferase (gammaTMT)* gene, complete cds	1690 bp
6.	OK031972.1	*Oryza sativa* Indica Group	Isolate RWG-405 *gamma-tocopherol methyl transferase (gammaTMT)* gene, complete cds	1690 bp
7.	XM_002454384.2	*Sorghum bicolor*	Probable tocopherol O-methyltransferase, chloroplastic (LOC8073167), mRNA	374 bp
8.	KF184802.1	*Saccharum* hybrid cultivar R570	Clone BAC 258H24 complete sequence	2092 bp
9.	JQ246243.1	*Zea mays* subsp. mays cultivar By804	*Gamma-tocopherol methyltransferase* gene, complete cds	1699 bp
10.	XM_062364039.1	*Phragmites australis*	Probable tocopherol O-methyltransferase, chloroplastic (LOC133919600), mRNA	368 bp
11.	AJ920394.2	*Triticum aestivum*	mRNA for g-TMT protein	309 bp

**Table 3:** BLAST results of *γ-TMT* gene sequences from mutant *T. flagelliforme*.

Species Name	Description	E-Value		Per. Identity		Acc. len	Accession
		KB 6-9-3	KB 6-3-3-6	KB 6-9-3	KB 6-3-3-6		
*Carthamus tinctorius* (family: Asteraceae)	*Gamma-tocopherol methyltransferase* (*γ-TMT*) gene, complete cds	8 × 10^−27^	1 × 10^−25^	77.90%	77.35%	3393 bp	HM028671.2
*Carthamus oxyacanthus* (family: Asteraceae)	*Gamma-tocopherol methyltransferase* (*γ-TMT*) gene, complete cds	8 × 10^−27^	3 × 10^−25^	76.97%	76.11%	3371 bp	JX035783.1
*Lactuca sativa* (family: Asteraceae)	Gamma-tocopherol methyltransferase mRNA, complete cds	8 × 10^−27^	3 × 10^−25^	76.67%	76.11%	1131 bp	FJ975047.1
*Helianthus annuus* (family: Asteraceae)	*Gamma-tocopherol methyltransferase* (*γ-TMT*) gene, complete cds	3 × 10^−20^	6 × 10^−22^	74.03%	74.59%	4029 bp	DQ229829.1
*Tabernaemontana elegans* (family: Apocynaceae)	Gamma-tocopherol C-methyltransferase (TMT) mRNA, partial cds	2 × 10^−22^	2 × 10^−21^	77.99%	77.36%	807 bp	PP067960.1
*Glycine max* (family: Fabaceae)	g-tmt mRNA for gamma-tocopherol methyltransferase, complete cds	3 × 10^−20^	1 × 10^−18^	74.16%	73.60%	1097 bp	AB617795.1
*Elaeis oleifera* (family: Arecaceae)	Gamma-tocopherol methyltransferase (g-TMT) mRNA, partial cds	3 × 10^−19^	4 × 10^−18^	75.00%	74.39%	1284 bp	EU057617.1
*Olea europaea* (family: Oleaceae)	Gamma-tocopherol methyltransferase (GTMT) mRNA, complete cds	3 × 10^−19^	1 × 10^−17^	76.71%	76.03%	1277 bp	KT777643.1

**Table 4:** Nucleotide mutation variations in the *γ-TMT* gene of mutant *T. flagelliforme* (KB 6-9-3 and KB 6-3-3-6) compared with the wild-type.

Position of Mutations in Mutants (bp)	Variation in Mutations Between Mutants and Wild-Type	Type of Mutations
533	C to G	Substitution
537	T to A	Substitution
544	GTG to CAA	Substitution
546	T to A	Substitution
556	A to G	Substitution
559	AGC to TCT	Substitution
568	GT to TG	Substitution
577	TA to GG	Substitution
580	A to G	Substitution
586	GT to AA	Substitution
591	G to A	Substitution
593	A to C	Substitution
596	TA to GC	Substitution
540	G	Deletion in wild-type
553	T	Deletion in wild-type
564	GA	Deletion in wild-type
574	CG	Deletion in wild-type

**Table 5:** Nucleotide mutation variations in the *γ-TMT* gene between *T. flagelliforme* mutants (KB 6-9-3 and KB 6-3-3-6).

Position of Mutations in Mutants (bp)	Variation of Mutations	Type of Mutations
337	T	Deletion in KB 6-9-3
374	T	Deletion in KB 6-9-3
356–357	CT to TC	Substitution
358–359	TC	Deletion in KB 6-9-3
607	T to C	Substitution
862	T to C	Substitution
971	A to G	Substitution
1051	G to A	Substitution

**Table 6:** BLAST protein results for *T. flagelliforme* mutant KB 6-9-3.

No	Accession	Organism	Protein Name	Identity	E-Value	Query Cover
1.	GAB2221792.1	*Drosera rotundifolia*	Hypothetical protein	73.61%	3 × 10^−24^	21%
2.	XP_018837640.1	*Juglans regia*	probable tocopherol O-methyltransferase	70.83%	1 × 10^−23^	21%
3.	KAJ3707098.1	*Rhynchospora tenuis*	Hypothetical protein	70.83%	1 × 10^−23^	21%
4.	EOY10208.1	*Theobroma cacao*	Gamma-tocopherol methyltransferase	73.61%	3 × 10^−23^	21%
6.	KAJ4799986.1	*Rhynchospora pubera*	Gamma-tocopherol methyltransferase	70.83%	4 × 10^−23^	21%
7.	KAJ4720949.1	*Melia azedarach*	Gamma-tocopherol methyltransferase	72.22%	5 × 10^−23^	21%
8.	XP_071685828.1	*Rutidosis leptorrhynchoides*	Gamma-tocopherol methyltransferase	72.22%	6 × 10^−23^	21%

## Data Availability

The data presented in this study are openly available in Figshare [https://doi.org/10.6084/m9.figshare.29085020, accessed on 16 May 2025].

## Supplementary Materials

The following supporting information can be downloaded at https://www.mdpi.com/article/10.3390/ijms26157148/s1.
