# Supplementary material for: Sequence Variation and In Silico Protein Characterization of γ-TMT Gene in Mutant Rodent Tuber (Typhonium flagelliforme Lodd.)
Source: Int J Mol Sci. 2025 Jul 24;26(15):7148. doi: 10.3390/ijms26157148 (PMC12346533; doi:10.3390/ijms26157148)
Supplement: Supplementary file 1 [file ijms-26-07148-s001.zip › ijms-3683660-supplementary.pdf]

Supplementary Materials

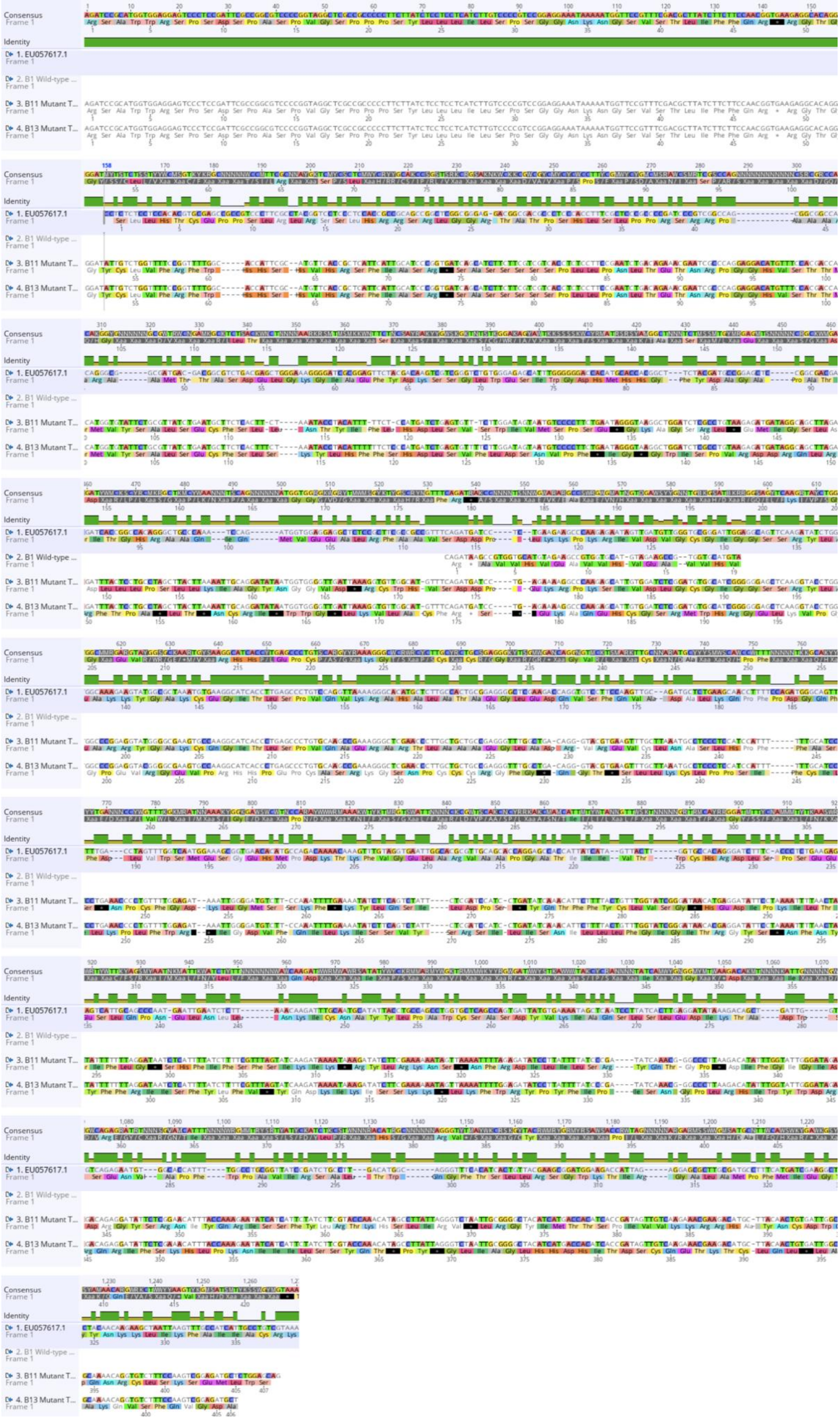

Figure S1. Amino acid translation of mutant and wild-type *T. flagelliforme* sequences based on the start codon. EU057617.1: *Elaeis oleifera* gamma-tocopherol methyltransferase (g-TMT) mRNA, partial coding sequence (used as reference for start codon). B1 (KB control): wild-type clone. B11 (KB 6-9-3), B13 (KB 6-3-3-6): mutant clones.

Compute pI/Mw - Results — A

Theoretical pI/Mw (average)

Sequence:

```
        10         20         30         40         50         60
CLVFRFWHHS HVHRSFIASR SASSSSSPLL PNLTETNRPG GHVSTTMVYS ALSECFSLLN

        70         80         90        100        110        120
TYIFLHDLSV SWIVMSPSEG KAGSRLEMIG SLDLLLPSLL KIAGYNGGVD RCWHVSDDPE

       130        140        150        160        170        180
KRPKSIVDLG CGIGGSSRYL ARRYGAKCQG ITLSPVQAER ARTLAAAEGL ADRVREVCLN

       190        200        210        220        230        240
ASLHPFFASN PCFGDKLGMS SKFKYLQSIL DPSYQTFFYC LVSGHEDIPK ILTIFLGSHF

       250        260        270        280        290        300
IFSFSIKIKR YLRKNSNFRD ILILSRYQTG PDIFGIGIDR GYSRNIYQRI SSFYLRTKHS

       310        320        330        340
LIRVLRGYIM TTSPIVVKKR RHAYNCDWQN RCLSKSEMLW S
```

Theoretical pI/Mw: 9.66 / 38533.47

Compute pI/Mw - Results — B

Theoretical pI/Mw (average)

Sequence:

```
        10         20         30         40         50         60
CLVFRFWHHS HVHRSFIASR SASSSSSPLL PNLTETNRPG GHVSTTMVYS ALSECFSLSK

        70         80         90        100        110        120
YLHFFSHDLS VFLDSNVPFI GGWISPVRDD RQLRFTPALT NCRIWWGLKV LACFRSEKAQ

       130        140        150        160        170        180
EHCGSRMWHR GELKVPGPEV RGEVPRHHPE PCASRKGSNP CCCRGFGQGT SLLKCLPPSI

       190        200        210        220        230        240
FCILKPLFWR IGDVFQILKI SSVYSRSILI SNILLLFGIG ITRGYSNFNY FFRIISFYLF

       250        260        270        280        290        300
VYQDKIKISS KKLKFWRYPY FIPISNGPLR HIWYWDRQRI FSKHLPKNII ILSSYQTPYG

       310        320        330
LIAGLHHDHI TDSCQETKTC LQLLAKQVSF QVGDA
```

Theoretical pI/Mw: 9.67 / 38565.77

Figure S2. Calculation of isoelectric point and molecular weight using Expasy. A: Mutant KB 6-9-3. B: Mutant KB 6-3-3-6. Red arrows: isoelectric point and molecular weight of the protein.
